# Clinical and economic burden of respiratory syncytial virus in children aged 0–5 years in Italy

**DOI:** 10.1186/s13052-024-01628-7

**Published:** 2024-03-25

**Authors:** Melania Dovizio, Chiara Veronesi, Fausto Bartolini, Arturo Cavaliere, Stefano Grego, Romina Pagliaro, Cataldo Procacci, Loredana Ubertazzo, Lorenzo Bertizzolo, Barbara Muzii, Salvatore Parisi, Valentina Perrone, Eugenio Baraldi, Elena Bozzola, Fabio Mosca, Luca Degli Esposti

**Affiliations:** 1 CliCon Società Benefit S.r.l., Health, Economics & Outcomes Research, Via Murri 9, Bologna, 40137 Italy; 2 USL Umbria 2, Dipartimento Farmaceutico, Perugia, Italy; 3 UOC Farmacia Aziendale, ASL Viterbo, Viterbo, Italy; 4 Dipartimento Tecnico-Amministrativo, ASL 3 Genovese, Genova, Italy; 5 UOC Farmaceutica Territoriale, Azienda Sanitaria Locale Roma 5, Rome, Italy; 6 Dipartimento Farmaceutico, ASL BAT (Barletta-Trani-Andria), Trani, Italy; 7 UOC Farmacia Territoriale, ASL Roma 4, Rome, Italy; 8 Sanofi Vaccines, Milan, Italy; 9 Neonatal Intensive Care Unit, Department of Woman’s and Child’s Health, Padova University Hospital, Padova, Italy; 10 Pediatric Disease Unit, Bambino Gesù Children Hospital IRCCS, Rome, Italy; 11 Department of Clinical Sciences and Community Health, University of Milan, Milan, Italy

**Keywords:** Children, Monoclonal antibodies, Infants, Prevention of RSV, Real-world evidence, Respiratory syncytial virus

## Abstract

**Background:**

Respiratory syncytial virus (RSV) is among the leading causes of hospitalization due to lower respiratory tract infections (LRTIs) in children younger than 5 years worldwide and the second cause of infant death after malaria. RSV infection occurs in almost all the infants before the second year of life with variable clinical severity, often requiring medical assistance. This analysis investigated patients aged 0–5 years with RSV infection focusing on epidemiology, clinical features, and economic burden of RSV-associated hospitalizations in a setting of Italian real clinical practice.

**Methods:**

An observational retrospective analysis was conducted on administrative databases of healthcare entities covering around 2.6 million residents of whom 120,000 health-assisted infants aged < 5 years. From 2010 to 2018, pediatric patients were included in the presence of hospitalization discharge diagnosis for RSV infections, and RSV-related acute bronchiolitis or pneumonia. Epidemiology, demographics, clinical picture and costs were evaluated in RSV-infected patients, overall and stratified by age ranges (0–1, 1–2, 2–5 years) and compared with an age-matched general population.

**Results:**

Overall 1378 RSV-infected children aged 0–5 years were included. Among them, the annual incidence rate of RSV-related hospitalizations was 175–195/100,000 people, with a peak in neonates aged < 1 year (689–806/100,000). While nearly 85% of infected infants were healthy, the remaining 15% presented previous hospitalization for known RSV risk factors, like preterm birth, or congenital heart, lung, and immune diseases. The economic analysis revealed that direct healthcare costs per patient/year were markedly higher in RSV patients than in the general population (3605€ vs 344€).

**Conclusions:**

These findings derived from the real clinical practice in Italy confirmed that RSV has an important epidemiological, clinical, and economic burden among children aged 0–5 years. While the complex management of at-risk infants was confirmed, our data also highlighted the significant impact of RSV infection in infants born at term or otherwise healthy, demonstrating that all infants need protection against RSV disease, reducing then the risk of medium and long-term complications, such as wheezing and asthma.

**Supplementary Information:**

The online version contains supplementary material available at 10.1186/s13052-024-01628-7.

## Background

Respiratory syncytial virus (RSV) is one of the leading causes of hospitalization due to lower respiratory tract infections (LRTIs) in children younger than 5 years worldwide, and the second cause of infant death after malaria [1, 2]. Globally, it has been estimated that RSV is responsible every year for 33 million cases of LRTIs requiring medical assistance at the outpatient level, 3.6 million hospitalizations and more than 100,000 deaths, of which more than 26,000 in-hospital [1]. RSV results in elevated mortality also in high-income countries, being 10-times greater than deaths from influenza (3.1 versus 0.3 deaths per 100,000 infants aged < 1 year) [3]. RSV is the most common etiologic agent identified in respiratory infections among young children, with almost 100% of children infected at least once by the age of 2 years and with a clinical severity that often needs medical assistance [1, 2]. Studies conducted in the US and in Europe indicated the higher vulnerability of the youngest children and confirmed that RSV infection is clinically more burdensome in infants up to 24 months old [4, 5]; one underlying reason might be that lungs and immune system are still developing during the first months of life [5, 6].

International and Italian data reported that among neonates and children at their first RSV season, about one-third of those who develop an RSV airway infection are managed in outpatient medical setting, while less than 10% have to access the emergency department to be then hospitalized, in some cases in intensive care unit (ICU) [7–10].

Furthermore, in children aged less than 12 months approximately 80% of bronchiolitis hospitalizations [11] and 40% of pneumonia hospitalizations [12] are due to RSV.

RSV is a seasonal virus; in Italy, the circulation of the virus starts between October and November, peaking in February, and ends in March–April, for a total duration of about 5 months [1].

All infants are at risk of RSV-LRTIs, because of two main risk factors for the disease that apply to all infants: seasonality and age < 1 year. Additional risk factors for RSV-LRTIs are preterm birth, congenital heart disease (CHD), chronic lung disease (CLD) or bronchopulmonary dysplasia (BPD), and other specific and severe diseases leading to immune and neuromuscular deficits [1, 13, 14]. Actually, as confirmed by recent Italian studies, almost 9 out of 10 infants hospitalized for RSV [10] and even more that 9 out of 10 infants with LRTIs visited at family pediatrician [15] were full-term born or otherwise healthy. This segmentation of the RSV-LRTIs burden retrieved in Italy is in line with the evidence available in the literature and studies from the Centers for Disease Control and Prevention (CDC) in USA [16–18].

Furthermore, RSV disease is often not self-limiting and might give long-term sequelae, as suggested by the fact that around 30–40% of children with prior bronchiolitis-related hospitalizations are likely to experience recurrent bronchospasm episodes and asthma as long-term complications [19].

Given the substantial clinical burden in terms of morbidity and mortality, RSV disease is recognized by the World Health Organization (WHO) as a global public health priority [20]. Unfortunately, at present there is no safe and effective antiviral or therapy available, and only supportive care is usually provided in the hospital setting [1, 21]. Regarding prevention, at present there is no approved vaccine to protect all infants from RSV disease. Indeed, two protein-based vaccines have both reached the phase III of clinical trials; however, in spite of the promising efficacy (from 86 to 94%), up to now they have been only tested in the elderly population [6].

Passive immunization with palivizumab, a short-acting monoclonal antibody (mAb), is the only currently licensed prophylaxis, but it is recommended only for infants at higher risk of RSV [14]. Recently, nirsevimab, a novel long-acting mAb was authorized by the European Medicines Agency to protect all infants at their first RSV season [22], in view of its satisfactory efficacy proved in pivotal clinical trials (about 80%) in reducing the incidence of RSV-related LRTIs [23], and further confirmed by preliminary results in a phase 3b real-world evidence study [24].

Given the major public health impact of RSV, the WHO [20], the European Centre for Disease Prevention and Control (ECDC) [25], as well as by the Italian Guidelines on RSV bronchiolitis management [26, 27] have has recognized nirsevimab as a promising preventive strategy, that might be shortly inserted into routine immunization calendars, to protect all newborns and infants entering in their first RSV season.

An understanding of the burden of RSV disease at a national level can be of value for informing health policy makers and to optimize RSV management and prevention. However, routinely collected data reporting information on healthcare consumptions are underutilized to evaluate RSV burden [28–30].

In this context, the present real-world analysis was aimed at analysing the epidemiology of RSV-associated hospitalizations, and the clinical and economic burden of patients up to 5 years of age with RSV infection, in Italian settings of clinical practice.

## Methods

### Data source

An observational retrospective analysis was performed on data collected from the following administrative databases of a sample of Italian healthcare entities accounting for approximately 2.6 million health-assisted subjects (comprising 120,000 health-assisted infants between 0 and 5 years of age): demographic database that contains patients’ demographic data such as age and gender; pharmaceuticals database providing data on drug prescriptions, as ATC (Anatomical Therapeutic Chemical) code, number of packages, number of units per package, unit cost per package, and prescription date; hospitalization database that includes all hospitalization data with discharge diagnosis codes classified according to the International Classification of Diseases, Ninth Revision, Clinical Modification (ICD-9-CM), Diagnosis Related Group (DRG) and DRG-related charge (provided by the Health System); outpatient specialist services database, which contains date and type of service delivered, description of diagnostic tests and visits, and charge for laboratory test or specialist visit.

To guarantee patients’ privacy, an anonymous univocal numeric code was assigned to each subject included in the study, in full compliance with the European General Data Protection Regulation (GDPR) (2016/679). The patient’s code in each database allowed the electronic linkage between all different databases. No identifiers related to patients were provided to the authors. All the results of the analyses were produced as aggregated summaries, impossible to be attributed, either directly or indirectly, to individual patients. Informed consent was waived due to the use of encrypted retrospective information for research purposes and because it is not required when obtaining it is impossible for organizational reasons (pronouncement of the Data Privacy Guarantor Authority, General Authorization for personal data treatment for scientific research purposes – n.9/2014). According to the Italian law [31], this study has been notified to and approved by the following local Ethics Committee of the participating healthcare bodies: “Comitato etico interprovinciale area 1″ (AOU Foggia, ASL FG, ASL BAT)”, Protocol N. 84/segCE/2019 of 05/11/2019; “Comitato Etico della Regione Liguria”, Protocol N. 0000510 of 16/10/2019; “Comitato Etico Lazio I”, Protocol N. 479/CE Lazio I of 14/06/2019; “Comitato Etico Lazio I”, Protocol N. 50/CE Lazio I of 10/01/2018; “Comitato Etico Regionale Umbria”, Protocol N. 17,844/19/ON of 28/11/2019; “Comitato Etico Lazio I”, Protocol N. 304/CE Lazio I of 13/02/2019.

### Study population

All patients aged 0 to 5 years were included from January 2010 to December 2018 (inclusion period) if they presented a diagnosis of RSV infections identified by hospitalization discharge diagnosis at primary or secondary level of RSV (ICD-9-CM code: 079.6) or acute bronchiolitis due to RSV (ICD-9-CM code: 466.11) or pneumonia due to RSV (ICD-9-CM code: 480.1). All assisted individuals with a change of Region/LHU (relocation) during the follow-up period, and patients over 5 years of age were excluded.

The index date corresponded to the first hospitalization for RSV during the inclusion period. Patients were observed for 12 months before (characterization period) and after the index date (follow-up period), thus the total observation period was between January 2009 to December 2019. Based on the date of birth (month and years or only years, if birthdate was not available), patients were stratified by age range: i) group 0–1 year, that included all children from birth to their first birthday (excluded); ii) group 1–2 years, that included all children from one year of age up to their second birthday (excluded); iii) group 2–5 years, that included all children from two years of age up to their fifth birthday (included). Moreover, during 2019 (which represented the most recent year of the study period), an age-matched general population (children aged 0–5 years) without RSV diagnosis was selected to carry out comparisons with a healthy control group.

### Study variables

#### Epidemiology

The annual incidence rate of RSV hospitalized individuals was defined as the number of newly hospitalized patients for RSV each year per 100,000 inhabitants (aged between 0 and 5 years). The incidence rate was reported for overall patients and for those stratified by age classes.

#### Evaluation of the most frequent treatments and hospitalizations, and underlying diseases

Considering the first year after RSV hospitalization, the presence of the most frequently prescribed treatments, grouped by first level ATC code, and the most common causes of hospitalization, grouped by major diagnostic category (MDC) were recorded in both RSV patients and general population. Moreover, the occurrence of hospitalization for underlying diseases [32–34], such as premature birth (under 37 gestational week) (ICD-9 CM code 765.10–765.19), congenital cardiopathy (ICD-9 CM Code 745.0–747.4), chronic lung disease (ICD-9 CM code 770.7), cerebral palsy (ICD-9 CM code 343.9), hematological diseases (ICD-9 CM code 200.2–205.9), and acute bronchiolitis due to other infectious organisms (ICD-9-CM 466.19), were evaluated during the characterization period. When data were available, also information concerning the type of hospital setting (day hospital, pediatric units, ICU) and the delivery of ventilation procedures [tracheostomy (ICD 9 CM 30.1–30.2), respiratory therapy (ICD 9 CM 93.9), other continuous invasive mechanical ventilation (ICD 9 CM 96.7), insertion of endotracheal tube (ICD 9 CM 96.04)], was assessed.

#### Healthcare direct costs covered by the Italian national health system

Total healthcare direct cost analysis was performed from the perspective of the Italian National Health System (INHS), with costs reported in Euros (€). Total costs comprised the expenses for drugs (evaluated using the INHS purchase price), hospitalizations (determined using DRG tariffs, which represent the reimbursement levels by the INHS to healthcare providers), and outpatient specialist services (defined according to tariffs applied by each region). The cost analysis reported the mean annual cost per patient for RSV patients as well as for the general population.

### Statistical analysis

A descriptive statistical analysis was conducted on continuous variables, presented as mean ± standard deviation (SD), and categorical variables, expressed as numbers and percentages. In compliance with the Italian Code for protection of personal data (“Codice in materia di protezione dei dati personali”, D. Lgs. 196/2003)” for subgroups with a numerosity below four patients, data were reported as not issuable (NI) for data privacy, given that the results might be potentially attributable to single individuals. All the analyses were performed using STATA SE v17.0 (StataCorp., College Station, TX, USA).

## Results

### Epidemiological data

**Fig. 1:**
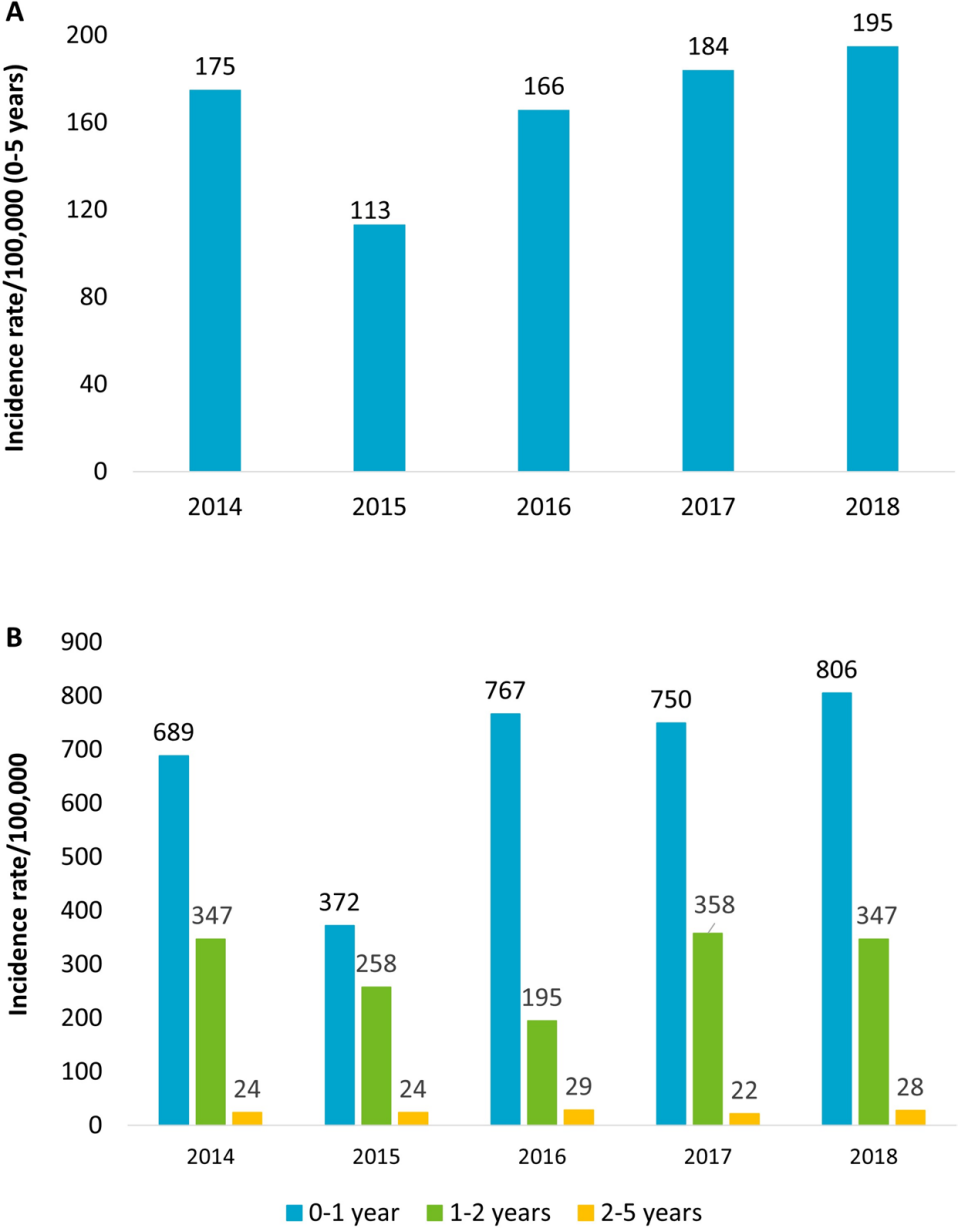
Annual incidence of RSV hospitalizations among the pediatric population, overall (0–5 years) (**A**) and stratified by age (**B**)

The annual incidence rate of RSV-related hospitalizations, evaluated across 2014 and 2018, ranged from 175 to 195 cases/100,000 among the pediatric population aged 0–5 years (Fig. 1A). The stratification of patients by age showed that the annual incidence rate of RSV-related hospitalizations was between 689 and 806 cases/100,000 in patients under 1 year of age, 347 cases/100,000 in those between 1–2 years, and 24–29 cases/100,000 among 2–5 years patients (Fig. 1B).

Using ICD-9-CM 466.19 to detect hospitalization for the acute bronchiolitis due to any infectious organisms in the general population during 2019, almost 2130 cases/100,000 individuals were identified, specifically 1024/100,000 in 0–1 years age range and 1099/100,000 in 1–2 years age range (data not shown).

### Characteristics of RSV-infected children and age-matched general population

From the overall 120,000 pediatric health-assisted subjects aged 0–5 years, 1378 (1.1%), patients with RSV diagnosis meeting the inclusion criteria were identified and compared with age-matched infants within the same sample population. Among RSV patients, 829 (60.2%) were in the 0–1 years age class, 406 (29.5%) and 143 (10.4%) in the 1–2 years and 2–5 years age classes, respectively.

During the characterization period, the occurrence of underlying diseases requiring hospital admission was estimated. Among total 1378 children aged 0–5 years infected with RSV, 1169 (84.8%) did not present any previous hospitalization for the risk factors of severe disease form considered in the present analysis, namely premature birth, congenital cardiopathy, chronic lung disease, cerebral palsy, hematological diseases, and acute bronchiolitis due to other infectious organisms. The occurrence of risky complications in the remaining RSV-infected patients (with reference to the general population without RSV infection) is reported in Table 1. In detail, 8.4% had a previous hospitalization for preterm birth defined by a gestational age below 37 weeks (compared to 0.5% in the general population), 4.3% for congenital cardiopathy (0.3% in the general population) and 3.6% for acute bronchiolitis due to other infectious organisms (0.3% in the general population). In all age classes, preterm birth was the most common cause of hospitalization prior the index date, specifically in 10.1%, 7.6% and 11.2% of the RSV-infected infants aged 0–1 year, 1–2 years and 2–5 years, respectively.

**Table 1:** Hospitalization for underlying diseases during the characterization period, in patients and in the general population, stratified by age. Data are given as number and percentages (in brackets) for RSV patients and percentages for the general population

	**RSV patients (*N* = 1378)**				**General population**			
**Description**	**Age 0–1 year** **(*N* = 829)**	**Age 1–2 years** **(*N* = 406)**	**Age 2–5 years** **(*N* = 143)**	**Age 0–5 years** **(*N* = 1378)**	**Age 0–1 year**	**Age 1–2 years**	**Age 2–5 years**	**Overall 0–5 years**
Premature birth (< 37 gw)	84 (10.1%)	31 (7.6%)	16 (11.2%)	116 (8.4%)	3.1%	0.1%	0%	0.5%
Congenital cardiopathy	33 (4.0%)	13 (3.2%)	13 (9.1%)	59 (4.3%)	1.7%	0.2%	0%	0.3%
Chronic lung disease	0 (0%)	NI	0 (0%)	NI	0%	0%	0%	0%
Cerebral palsy	0 (0%)	0 (0%)	0 (0%)	0 (0%)	0%	0%	0%	0%
Hematological diseases	0 (0%)	0 (0%)	0 (0%)	0 (0%)	0%	0%	0%	0%
Acute bronchiolitis due to other infectious organisms	24 (2.9%)	19 (4.7%)	6 (4.2%)	49 (3.6%)	1.0%	1.1%	0%	0.3%

### Clinical history

The clinical burden of RSV patients was also evaluated by assessing the use of medications and the recurrence of hospitalizations during the first year of follow-up. As reported in Table 2, in RSV patients the most prescribed drugs were systemic antibacterials (72.4%, compared to 50.7% in the general population), drugs for obstructive airway diseases (70.1%, compared to 29.8% in the general population), and corticosteroids (20.5%, compared to 14.5% in the general population).

**Table 2:** Most frequently prescribed drugs during the first year of follow-up in RSV patients and in the general population. Data are given as number and percentages (in brackets) for RSV patients and percentages for the general population

ATC code	Description	RSV patients (*N* = 1,378)	General population
J01	Antibacterials for systemic use	998 (72.4%)	50.7%
R03	Drugs for obstructive airway diseases	966 (70.1%)	29.8%
H02	Corticosteroids for systemic use	282 (20.5%)	14.5%
A11	Vitamins	106 (7.7%)	4.2%
A02	Drugs for acid related disorders	59 (4.3%)	0.5%

The analysis replicated in children stratified by age classes confirmed the same trends observed in the overall population (Supplementary Table S1). Regardless of age, the most common prescriptions in RSV patients at one-year follow-up were antibacterials for systemic use, drugs for obstructive airway diseases, systemic corticosteroids, vitamins, and drugs for acid-related disorders (antacids and drugs for peptic ulcer and gastro-oesophageal reflux disease). All of them showed a higher utilization in RSV group than in the general population. In the age-matched children without RSV infection, the largest proportion of treatments included antibacterials for systemic use and drugs for obstructive airway diseases. Table 3 details the most frequent hospitalizations during the first year of follow-up: 9.6% of RSV patients had at least one hospitalization related to respiratory system (1.3% in the general population), 3.2% (0.6% in the general population) due to other factors influencing health status (not specified), 2.6% (0.7% in the general population) and 2.1% (0.6% in the general population) due to digestive system and other infections, respectively.

**Table 3:** Most frequent causes of hospitalizations in RSV patients during the first year of follow-up and in the general population. Data are given as number and percentages (in brackets) for RSV patients and percentages for the general population

Major Diagnostic Category (MDC) – Description	RSV patients (*N* = 1378)	General population
Respiratory system	132 (9.6%)	1.3%
Other factors influencing health status	44 (3.2%)	0.6%
Digestive system	36 (2.6%)	0.7%
Infectious and parasitic diseases	29 (2.1%)	0.6%
Diseases and disorders of the endocrine, nutritional and metabolic systems	29 (2.1%)	1.0%

### Type of hospital admission

The clinical burden was also evaluated considering the type of clinical setting at hospital admission, in the period from RSV hospitalization up to the following three years. Table 4 details the distribution of type hospital admissions (day hospital, pediatric units and ICU), delivery of ventilation procedure in overall RSV patients and stratified by age classes. It should be specified that the sum of the percentages is not 100%, since each child might have required more than one type of hospitalization or procedure. Based on data availability, 18.7% of patients had at least one hospitalization in day-hospital setting, 32.9% in pediatric units, 2.9% in ICU. Moreover, 77.6% of patients underwent at least one ventilation procedure. The length of hospital stay (mean ± SD) was 8.5 ± 17.6 days for overall RSV patients, and 7.0 ± 11.3, 10.2 ± 24.2, and 11.9 ± 23.5 in 0–1 year, 1–2 years and 2–5 years age groups, respectively.

**Table 4:** Percentage of RSV patients (by age classes and overall) with at least one hospitalization during 3-year follow-up period, divided by type of type of hospital setting (Day Hospital, Pediatric Units, and ICU) and the delivery of ventilation procedures. Data are given as number and percentages (the sum of the percentages is not 100%, since each child might have required more than one type of hospitalization or procedure)

	**Age 0–1 year** **(*N* = 829)**	**Age 1–2 years** **(*N* = 406)**	**Age 2–5 years** **(*N* = 143)**	**Age 0–5 years** **(*N* = 1378)**
Day hospital	88 (15.7%)	47 (17.2%)	40 (39.2%)	175 (18.7%)
Intensive care unit	18 (5.0%)	NI	0 (0%)	21 (2.9%)
Pediatric unit	173 (48.2%)	45 (17.0%)	20 (20.2%)	238 (32.9%)
Ventilation procedures	428 (76.4%)	225 (82.1%)	73 (71.6%)	726 (77.6%)

### Cost analysis

**Fig. 2:**
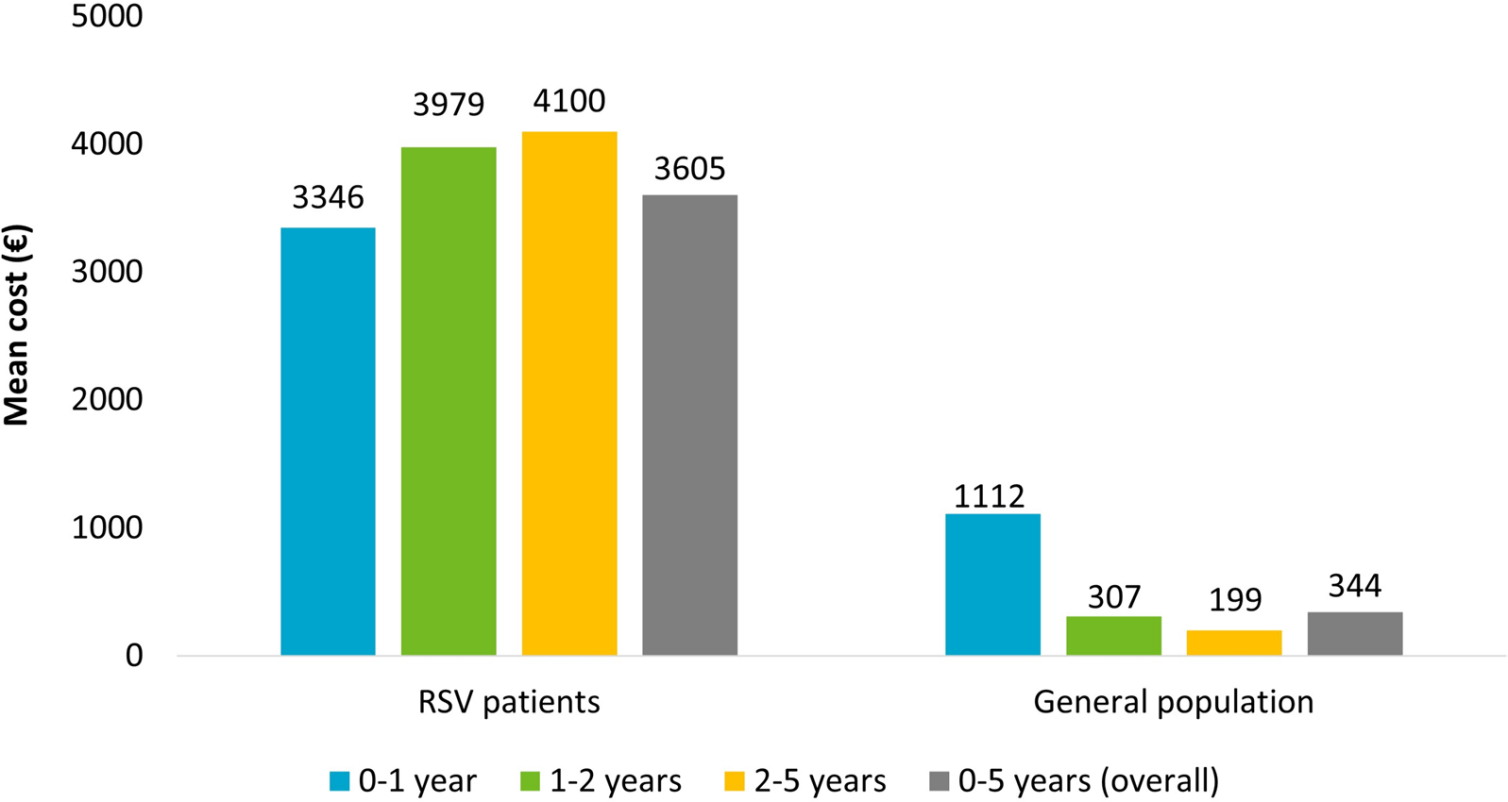
Mean annual costs/patient for the management of RSV during the first year of follow up period (index hospitalization included)

From RSV hospitalization up to one year after, the total direct healthcare costs covered by the INHS were estimated. As reported in Fig. 2, in overall RSV patients, the total annual cost per patient averaged 3605€ (compared to 344€ for the general population); specifically, 3346€ for 0–1 year patients, 3979€ for 1–2 years and 4100€ for 2–5 years RSV patients.

## Discussion

This administrative claims-based observational analysis provided insights into the epidemiology, the clinical and economic burden of RSV-hospitalized children in a real-world Italian setting.

Among 1378 RSV patients aged 0–5 years included from 2014 and 2018, the yearly RSV-related hospitalizations showed a peak of incidence rate in infants aged less than 1 year, confirming that being immunologically naïve to RSV exposure represents one main risk factor for RSV infection [1, 4, 32]. These data are line with previous Italian findings by Kuhdari and colleagues who reported that the proportion of RSV-hospitalized patients reached 88.8% in infants under 1 year of age, in front of 30% for the age range 1–2 years and of 10% for the age range 2–5 years [7]. However, the incidence rate of RSV infections found in our sample appeared to be lower compared to that previously described in other countries, with same epidemiology trend and socio-economic factors: in fact, hospitalization for RSV respiratory infection across high-income countries in the northern hemisphere is up to 4% of the entire cohort of children in their first RSV season, with up to 20% of them requiring ICU access [5, 8, 9, 28]. Recent data from five locations in different high-income European countries confirmed that acute respiratory infection due to RSV-associated causes resulted in the necessity of hospitalisation of one in every 56 healthy full term-born infants [35].

This inconsistency between RSV incidence in different national datasets might be explicated by the different periods of data availability and possibly also diagnosis coding bias, owing to time shift across countries in the transition from ICD9-CM to ICD-10 coding systems [36]. Although the present analysis was not aimed at classifying all the specific pathogen-coded admissions other than RSV, a uniform disease coding system represents a desirable achievement for future epidemiological studies to properly identify the causative etiologic agent of pediatric respiratory diseases [36]. Another possible explanation behind these discrepancies is reasonably climate. Our results showed a decrease in RSV hospitalization rate during 2015. This phenomenon might be due to the fact that 2015 was recorded as one of the warmest years [37], and this might have mitigated the outbreak of RSV infection [10, 38, 39]. In addition, 2015 was also characterized by a peak of influenza, which mainly affected the pediatric population (26.0% in the 0–4-year-old class) [40] and perhaps led to a hospitalization misdiagnosis [39]. Hence, it is possible that the burden of RSV hospitalization could be underestimated among our sample population.

In agreement with global and Italian evidence, the present analysis confirmed that the largest majority of RSV-associated infections necessitated some kind of medical assistance, namely day-hospital, ordinary hospitalization, admission to pediatrics unit, to ICU or interventions with ventilation procedures. The strikingly elevated proportion of patients who required ventilation procedures in all age intervals and in the overall RSV-infected infants, even higher than the percentage of ICU admission, might be explicated by the common practice of transferring the ventilators from the ICUs to sub-intensive or ordinary units in an effort to better manage the limited beds in intensive care and reserve them to the most critical patients. Of note, the requirement for ICU admission was markedly more frequent in the newborns aged less than 1 year (5%) than in the overall population of children aged 0–5 years (2.9%). Nevertheless, these numbers are anyhow lower that those reported in similar Italian studies. Barbati et al. described the epidemiology of RSV-related hospitalization between September 2014 and August 2019 in children aged 0–6 years, reporting an overall rate of admissions to ICU of 16.5%, mostly detected in the neonates below 1-year of age (86.4%) [10]. The divergence with our data might lie in the different study design and inclusion criteria. While the analysis by Barbati and colleagues was conducted in a single Italian region (Tuscany) and the patients’ selection was based on laboratory diagnosis (molecular testing on nasopharyngeal swab or bronchoalveolar lavage fluid or both), here we used ICD-9-CM code at hospitalization discharge as proxy of diagnosis, thus some cases might be missed (i.e. for unavailability of diagnostic test results data or miscoding hospitalization issues within the administrative databases). Moreover, the catchment area of health-assisted individuals of this analysis was represented by different regions from north, central, and south Italy.

In spite of the abovementioned discrepancies of our data with respect to other Italian and international incidence rates for RSV disease, the general emerging message is the increased susceptibility to severe infection (close to 60%) and the higher rate of RSV-related hospitalizations within the first year of life, consistent with previous national and international reports [5, 8, 9, 25]. In Italy, a study conducted by Kudhari and colleagues reported a proportion of 89% children aged below 1 year among 57,656 hospital admissions for RSV infection between 2001 and 2014 [7]. Consistently, more recent Italian data described a larger need for intensive care associated with younger children, with nearly 71% of offspring below three months [10]. The significant clinical burden of RSV infection for younger infants has been further corroborated by recent US data that estimated a yearly number of 472,000 admissions in emergency unit and 1.6 million pediatric practice visits attributed to RSV infections within 2 years after birth [4].

RSV infection has been shown to be the underlying cause for 80% of bronchiolitis hospitalizations [11] and 40% of pneumonia hospitalizations [12] in infants aged below 12 months. We showed that among the general population, during 2019, about 1% of neonates during their first year of life were assigned with the hospitalization code for acute bronchiolitis due to other infectious organisms. However, this finding should be interpreted with caution, for possible underdiagnosis of RSV as etiologic agent in case of missing test. It has been reported that among bronchiolitis cases identified by this code (acute bronchiolitis due to other infection organisms), 72.7% were non-tested episodes and 27.3% were tested; among tested cases, 45.6% were RSV positive cases [40]. The nature of RSV disease is unpredictable, and it is challenging to identify in advance which children will develop severe disease [41]. Climatic, seasonal and air quality conditions are known to have a relevant impact on RSV disease dynamics [42].

The current view of considering newborn without risk factors as ineligible for the available standard prophylaxis should deserve reconsideration: our data, as well as evidence from existing literature [16–18], seem to indicate that all neonates and infants (not only those with risk factors) entering in their first season need protection against RSV, regardless their gestational age at birth and underlying health conditions, and regardless their month of birth. Hence, a universal approach to prophylaxis embracing also healthy infants born at term or late preterm might be helpful to keep under control the risk of acute RSV infections, medium- and long-term complications, including recurrent wheezing and asthma during infancy, which in turn may require further hospitalizations and medication use [43, 44].

The inclusion period of the present study ended in December 2018, so we cannot evaluate the impact of COVID pandemic on RSV management. The trends of circulation of respiratory viruses, including RSV, registered during the past and the current seasons show how important is having timely and accurate surveillance to properly support public health decisions. While there is a need to strengthen both hospital and outpatient surveillance systems, prevention of RSV in all neonates and children may play a key role to contain RSV outbreak in this age group, also avoiding healthcare service disruption at both outpatient and hospital settings, like it has happened during the recent seasons in view of the huge health emergency of COVID pandemic [45]. Moreover, during the latest period of COVID-19 pandemic, a striking increase in RSV diagnoses has been observed in various countries. This unexpected resurgence might be explained by several factors, including the return to social activities for children, especially after mass vaccination campaigns [45, 46].

To further characterize the clinical burden of RSV infections, we focused on the most frequent prescribed treatments and causes of hospitalizations during the first year of follow-up. Almost 70% of patients received systemic antibacterials and drugs for obstructive airway diseases, while 20% were treated with systemic corticosteroids. Nearly 10% of patients with an RSV hospitalization incurred in a second hospitalization during the first following year, due to respiratory system distress. Unsurprisingly, drug utilization and hospitalizations, regardless of the cause, were remarkably lower in the general population.

The economic burden in Italy for RSV patients has been investigated by Bozzola et al. on 531 patients from the Bambino Gesù Children Hospital of Rome that estimated an average annual cost per patient above 5750€ for RSV-related hospitalizations [47]. In the current analysis, we evaluated the total mean annual healthcare costs during the first year of follow-up, estimating 3605€ for RSV patients, compared to 344€ for the general population. In a recent systematic review for inpatient RSV management, the mean cost per patient was estimated as €4712 (95% CI, 4568–4856; range, €92 to €165,602).

The results must be interpreted with caution in view of some limitations related to its observational nature and the collection of data from administrative databases. Our cohort of patients reflected the real clinical practice by evaluating data from a subset of health-assisted individuals extracted from administrative databases of healthcare entities of Liguria, Puglia, Lazio, Umbria regions. In addition, the use of administrative data might provide lacking or limited clinical information on some confounders potentially influencing the results. The number of RSV patients could be underestimated due the fact that the diagnostic test results data were not available and due to a miscoding hospitalization issue. Moreover, the stratification of patients by age could be influenced by the fact that only for a subset of entities there were data available regarding the month and year of birth.

## Conclusion

Our data obtained from the real-world clinical Italian practice reported the epidemiology of RSV cases among children under 5 years old and showed that the disease has a significant impact on the clinical and therapeutic management and economic burden. An important message emerging from the present analysis is that, in front of the necessary attention on infants with risk factors, RSV infection represents an important concern also for those born at term or otherwise healthy. Hence, protection against RSV disease represents a desirable achievement shared from all children in an effort to reduce the risk of medium and long-term complications, such as wheezing and asthma.

### Supplementary Information

**Supplementary Material 1.**

## Data Availability

All data used for the current study are available upon reasonable request to CliCon S.r.l. Società Benefit, which is the body entitled to data treatment and analysis by the involved healthcare bodies.

## Abbreviations

ATC: Anatomical therapeutic chemical
BPD: Bronchopulmonary dysplasia
CHD: Congenital heart disease
CLD: Chronic lung disease
DRG: Diagnosis related group
ECDC: European centre for disease prevention and control
EMA: European medicines agency
FIMMG: Italian federation of general practitioners
FIMP: Italian federation of family pediatricians
GDPR: General data protection regulation
ICD-9-CM: International classification of diseases, ninth revision, clinical modification
ICU: Intensive care unit
INHS: Italian national health system
LHUs: Local health units
LRTIs: Lower respiratory tract infections
MDC: Major diagnostic category
NI: Not issuable
NITAGs: National immunization technical advisory groups
RSV: Respiratory syncytial virus
SIN: Italian society of neonatology
SIP: Italian society of pediatrics
SITI: Italian society of hygiene and preventive medicine
WHO: World health organization
