# Supplementary material for: Clinical and economic burden of respiratory syncytial virus in children aged 0–5 years in Italy
Source: Ital J Pediatr. 2024 Mar 25;50:57. doi: 10.1186/s13052-024-01628-7 (PMC10964524; doi:10.1186/s13052-024-01628-7)
Supplement: Supplementary file 1 — Supplementary Material 1. [file 13052_2024_1628_MOESM1_ESM.docx]

**SUPPLEMENTARY MATERIAL**

**Clinical and economic burden of Respiratory Syncytial Virus in children aged 0-5 years in Italy**

**Supplementary Table S1.** Most frequently prescribed drugs in included patients during the first year of follow-up and in the general population, stratified by age classes. Data are given as number and percentages (in brackets) for RSV patients and percentages for the general population.

|  |  | **RSV patients** | | | **General population** | | |
| --- | --- | --- | --- | --- | --- | --- | --- |
| **ATC code** | **Description** | **Age 0-1 years** | **Age 1-2 years** | **Age 2-5 years** | **Age 0-1 years** | **Age 1-2 years** | **Age 2-5 years** |
| J01 | Antibacterials for systemic use | 556 (67.1%) | 321 (79.1%) | 121 (84.6%) | 17.9% | 53.4% | 56.6% |
| R03 | Drugs for obstructive airway diseases | 536 (64.7%) | 323 (79.6%) | 107 (74.8%) | 17.1% | 38.3% | 30.5% |
| H02 | Corticosteroids for systemic use | 179 (21.6%) | 75 (18.5%) | 28 (19.6%) | 6.7% | 18.4% | 15.2% |
| A11 | Vitamins | 80 (9.7%) | 24 (5.9%) | NI | 6.9% | 9.5% | 2.5% |
| A02 | Drugs for acid related disorders | 30 (3.6%) | 21 (5.2%) | 8 (5.6%) | 0.7% | 0.7% | 0.4% |

NI, not issuable for privacy (<4 patients)
